# Prescriptions and Dispensing

**Published:** 1908-07-25

**Authors:** 


### THERAPEUTICS.

### PRESCRIPTIONS AND DISPENSING.

### PILLS.

Coating (*continued*).

Several formulæ for pill varnishes are in use. They are either solutions of sandarach in alcohol, alcohol and ether, or chloroform and ether, or they are made by macerating balsam of tolu in ether after the balsam has been used for making syrup of tolu. One part of spent balsam of tolu in three parts by measure of ether is a suitable strength, the undissolved portion being separated by decantation. For sandarach varnishes one or other of the following formulæ may be used:—

1.

| | |
|---|---|
| Sandarach ... ... ... ... | 4½ parts |
| Chloroform ... ... ... ... | 4 parts |
| Ether meth. (specific gravity 0.717) | 10 parts |

2.

| | |
|---|---|
| Sandarach ... ... ... ... | 1 part |
| Absolute alcohol ... ... ... | 2 fl. parts |
| Ether ... ... ... ... ... ... | 1 fl. part |

3.

| | |
|---|---|
| Sandarach ... ... ... ... | 1 part |
| Alcohol ... ... ... ... ... | 2 fl. parts |

A varnish made with ether will, of course, dry most quickly, and one in which alcohol alone is the solvent most slowly. When the solvent is likely to have any effect on anything in the pill itself, the most rapid-drying varnish is, of course, to be preferred.

The method of varnishing is extremely simple. The pills, which must not have any powder on their surface, are put into a covered pot, a little varnish added—usually two or three drops to a dozen fair-sized pills—and the pills first shaken and then rotated in the pot for a few moments. They are then turned out on to a tile or plate, separated from one another with the least touching possible, and allowed to dry. Before they are quite dry they should be moved about a little by giving a slight rotatory movement to the plate. The spot which has been in contact with the plate during drying is then not apparent. This method is preferable to the older fashion of impaling each pill upon a pin, and then dipping it bodily into the varnish; the pin-hole was apt to escape being varnished, so that air gained access not only to the surface of the pill, but actually into its interior.

*Silver-coated Pills.*

When pills which are to be silver-coated, for appearance' sake, contain any ingredient capable of acting upon silver, they must be varnished before coating. The application of the silver-leaf may be carried out in a covered pot, or in one of the box-wood "silver-coaters" supplied for the purpose. One silver leaf of the ordinary size is usually required for each six 5-grain pills, and the leaf is put into the pot or box first. The pills are then shaken in another pot with a few drops of dilute acacia mucilage till every part of the surface of each is moistened with it, using the least quantity of mucilage that will suffice. The pills should now be turned into the pot containing the silver. This pot is closed and rotated, the whole being smartly shaken once or twice to ensure that the pills are all separated from one another. The rotatory movement is continued until the pills are uniformly coated.

*Pearl-coated Pills.*

A perfect pearl-coating can only be obtained when working on a much larger quantity of pills than are usually made specially for any particular single patient; but with a little care and perseverance very good results can be obtained, even with small quantities. A tin or copper vessel with a rounded bottom is needed, and such vessels are supplied for the purpose.

If one of these is not obtainable, a large covered pot will serve. Talc, otherwise known as French chalk, is the material of the coating, and only the very finest powder ("subtiliss.") is suitable. A small quantity of this powder is placed in the coating vessel, and the pills are shaken into another pot containing weak mucilage; about three or four times the amount of the latter is required as was recommended for silver-coating. The moistened pills are then thrown into the talc and the vessel steadily rotated. Small additions of the fine talc are made at intervals of one or two minutes until inspection shows that a sufficient thickness of coating has been taken up. Rotation is continued for a little longer to polish the surfaces, and a final burnish is given by putting the coated pills into a long flannel bag something like a stocking, and running them from end to end a few times.

*Gelatin-coating.*

A thin layer of gelatin forms a suitable protection in many cases, and, being transparent, it does not change the appearance of the pill except by giving it a glazed surface instead of a dull one.

Various formulæ are in use for the gelatin solution that is used, the following being probably the one most frequently employed:—

| | |
|---|---|
| Gelatin ... ... ... ... ... | 4 oz. |
| Gum Acacia ... ... ... ... | 1 oz. |
| Boric Acid ... ... ... ... ... | ¼ oz. |
| Water ... ... ... ... ... | 2 pints |

The gum and the gelatin should be soaked in the water for some hours, and then dissolved with the aid of gentle heat. A water-bath should be used, to prevent burning. The boric acid is then added, and the whole strained if necessary. The mixture forms a solid mass when cold, and it is melted on the water-bath for use.

The pills to be coated are stuck on the points of needles, the eye-ends of the latter being fixed in corks, which serve as handles. A dozen or more needles can be fixed in one large cork. The pills having been impaled upon the needles, are dipped into the melted jelly and withdrawn. They are held turned downwards till a drop of the surplus liquid forms on each, and these drops are then removed by just touching the surface of the main bulk of liquid with them. The cork, with needles and pills, is then turned the other way up, and the coating left to dry.

Gelatin coating on a commercial scale is not carried out with the help of needles, but machines are used, in which the pills are held against small tubes by the generation of a partial vacuum behind them, and then dipped only half-way into the gelatin solution. When the coating has dried, the pills are held in a similar way by the coated side, and the other half is then dipped.

*Sugar-coated Pills.*

Sugar-coating cannot be satisfactorily performed on a small scale, so that a description of it would not be in place here.

Our next article will begin to deal with powders.

(*To be continued.*)

